# Applying Patient and Health Professional Preferences in Co-Designing a Digital Brief Intervention to Reduce the Risk of Prescription Opioid–Related Harm Among Patients With Chronic Noncancer Pain: Qualitative Analysis

**DOI:** 10.2196/57212

**Published:** 2025-04-25

**Authors:** Rachel A Elphinston, Sue Pager, Farhad Fatehi, Michele Sterling, Kelly Brown, Paul Gray, Linda Hipper, Lauren Cahill, Maisa Ziadni, Peter Worthy, Jason P Connor

**Affiliations:** 1 RECOVER Injury Research Centre The University of Queensland Herston Australia; 2 National Health and Medical Research Council Centre for Research Excellence - Better Health Outcomes for Compensable Injury The University of Queensland Brisbane Australia; 3 School of Psychology The University of Queensland Brisbane Australia; 4 Addiction and Mental Health Services Metro South Health Hospital and Health Service Brisbane Australia; 5 Health Equity & Access Unit Metro South Hospital and Health Service Brisbane Australia; 6 Centre for Health Services Research The University of Queensland Brisbane Australia; 7 School of Psychological Sciences Monash University Brisbane Australia; 8 Gallipoli Medical Research Foundation Greenslopes Private Hospital Brisbane Australia; 9 Faculty of Medicine The University of Queensland Brisbane Australia; 10 Tess Cramond Pain and Research Centre Metro North Hospital and Health Service Brisbane Australia; 11 School of Medicine Department of Anesthesiology, Perioperative, and Pain Medicine Stanford University Stanford, CA United States; 12 School of Health and Rehabilitation Sciences The University of Queensland Brisbane Australia; 13 National Centre for Youth Substance Use Research The University of Queensland Brisbane Australia

**Keywords:** chronic noncancer pain, CNCP, prescription opioid use, brief intervention, brief psychological intervention, co-design, patient partners, qualitative research, digital health

## Abstract

**Background:**

Few personalized behavioral treatments are available to reduce the risk of prescription opioid–related harm among patients with chronic noncancer pain.

**Objective:**

We aimed to report on the second phase of the co-design of a digital brief intervention (BI) based on patient and health professional preferences.

**Methods:**

Eligible patients with chronic noncancer pain (n=18; 10 women; mean age 49.5, SD 6.91 y) from public hospital waitlists and health professionals (n=5; 2 women; mean age 40.2, SD 5.97 y) from pain and addiction clinics completed semistructured telephone interviews or participated in focus groups exploring BI preferences, needs, and considerations for implementation. Grounded theory was used to thematically analyze the data.

**Results:**

We identified 5 themes related to intervention content from patient reports: relevance of the biopsychosocial model and need for improved awareness and pain psychology education; nonpharmacological strategies and flexibility when applying coping skills training; opioid use reflection and education, with personalized medication and tapering plans; holistic and patient-inclusive assessment measures and feedback; and inclusion of holistic goals targeting comfort and happiness. Five themes related to the process and guiding principles were identified: therapist guided; engaging features; compassionate, responsive, person-centered care; a digital solution is exciting, maximizing reach; and educate and normalize system and policy challenges. Finally, 5 themes were reflected in the health professionals’ reports: digital health use is rare but desired; digital health is useful for patient monitoring and accessing support; patient motivation is important; a digital BI app is likely beneficial and at multiple care points; and safe medication use and managing pain goals. The reported barriers from health professionals were intervention intensity, potential costs, and patient responsiveness; factors facilitating the implementation were the alignment of digital BIs with clinical models, a stepped-care approach, and feedback.

**Conclusions:**

This co-design study identified key content areas, guiding principles, enabling factors, and barriers from both patients and health professionals to guide the development of digital BIs. The knowledge gathered should inform future iterations of co-designing digital BIs for the population most at risk of the harmful effects of opioid medications.

## Introduction

Clinical guidelines recommend behavioral and nonpharmacological treatments and interdisciplinary rehabilitation for the treatment of chronic noncancer pain (CNCP) (eg, Dowell et al [1]). However, there is a substantial gap between research evidence and clinical practice, referred to as one of the “valleys of death” [2] in the management of CNCP. Barriers to patients accessing best practice, multidisciplinary, specialist pain care include long waiting times, geography, high demand for treatment, treatment intensity, lack of trained behavioral therapists, and cost [3-7]. Psychological treatments rarely reach patients with CNCP. Our research on patients’ lived experience has found that most patients with CNCP are not offered psychological treatment (eg, cognitive behavioral therapy) and are unaware that it is recommended as an effective first line treatment for pain [8]. These experiences are supported by national research, which shows that 87% of Australians with CNCP had not seen a psychologist for CNCP and almost 60% were not aware of the role of psychologists in pain care [9]. This is not unique to Australia. Patients around the world are not offered or referred to nonpharmacological interventions, particularly when receiving opioid therapy [4,10]. Increasingly high demand for psychologists and specialist pain clinicians [11,12] further complicates research translation.

Digital health interventions (DHIs) for pain have been highlighted as a potential innovative solution to the treatment accessibility problem, the growing burden of CNCP, and global health threats [13-15]. DHIs for pain provide potential advantages to health systems, providers, and patients such as improved accessibility, efficiency, and cost-effectiveness, but DHIs are affected by similar knowledge-to-practice gaps with added challenges in data privacy, security, and formulating regulatory guidelines and approvals. Patient access to DHIs outside of research studies is limited [16], reducing their real-world impact. Few DHIs that are publicly available have received empirical evaluation [16,17]. Bridging these gaps in traditional and digital pain care is a priority.

A lack of patient and health professional partnering in research has been cited as a central reason for research failing to translate into clinical practice [18-21]. Co-designing new DHIs has been highlighted as a potential solution to the translational challenge. Co-design involves meaningful stakeholder (eg, patients and health professionals) involvement in the design, implementation, and translation of research [22]. Engaging patient partners alongside health professionals in the co-design of DHIs allows for improved tailoring to individual preferences and needs, which has the potential to increase treatment effectiveness, acceptance, and adoption in practice.

Patients and health professionals have rarely been involved in the co-design of DHIs for CNCP since it was recommended more than a decade ago [23]. Most DHIs are developed by the for-profit software industry [24] and lack a theoretical framework [17]. Only 19% of the studies involved end users in development, and this is usually done in an ad hoc manner [25]. Involvement of health professionals in designing DHIs varies considerably (8.2% and 30.6%) [17,25]; few enable communication with health professionals or clinician access to patient data [25,26]; and almost none (<3%) involve both patients and health professionals [25].

There is an opportunity to optimize the research to practice nexus using co-design methods in all phases of developing new, brief DHIs. This study is part of a larger program of work to co-design, co-develop, and evaluate the feasibility of a digital brief intervention (BI) for patients with CNCP to reduce prescription opioid–related harm. Digital BIs provide a potentially efficient and scalable psychological and behavioral treatment option that facilitates rapid access to pain care [13,27]. In the first phase of the digital BI co-design, we examined the patients’ lived experiences of CNCP management with a particular focus on opioid therapy in individual interviews [8]. In this second phase, we advanced this body of work by examining the needs and preferences of patients and health professionals as well as implementation barriers and enablers. The knowledge gathered will continue to inform future steps in the co-design of digital BIs for the population most at risk of the harmful effects of opioid medications.

## Methods

### Participants and Recruitment

Eligible participants were patients diagnosed with a CNCP condition who were purposively sampled to ensure adequate representation of both sexes, with culturally and linguistically diverse backgrounds, and current and past use of opioid therapy. A full summary of participant recruitment is provided in a previous study [8]. In short, the patient eligibility criteria included the following: those aged between 18 and 70 years, experiencing clinical levels of CNCP (scoring ≥4 out of 10 on the Pain Numerical Rating Scale on average over the past week), and seeking treatment from public health specialist addiction or pain services. The patient exclusion criteria were as follows: high levels of distress (based on patient scores ≥13 on the Kessler Psychological Distress Scale [28] and follow-up telephone risk assessment or clinical judgment from the addiction specialist), non-English speaking, a history of recent injecting drug use, or currently tested positive with SARS-CoV-2.

Patients were recruited through a tertiary hospital specialist pain clinic or community addiction specialist service. Patients were either engaged by mailing an information pack inviting those on the waitlist to participate (pain clinic) or invited by the medical specialist and a member of the research team (RAE) during their on-site appointment (addiction service). The final sample size was determined by thematic saturation using the analysis approach by Guest et al [29] applied during the data collection and inductive thematic analysis phase [8].

Health professionals were also invited to participate in one-on-one interviews or focus groups to assess their needs and preferences for the digital BI. Health professionals recruited through the specialist pain and addiction clinics as well as through the research and clinical networks of the research team were eligible to participate if they were working in chronic pain management (public or private sector).

### Design

**Figure 1:**
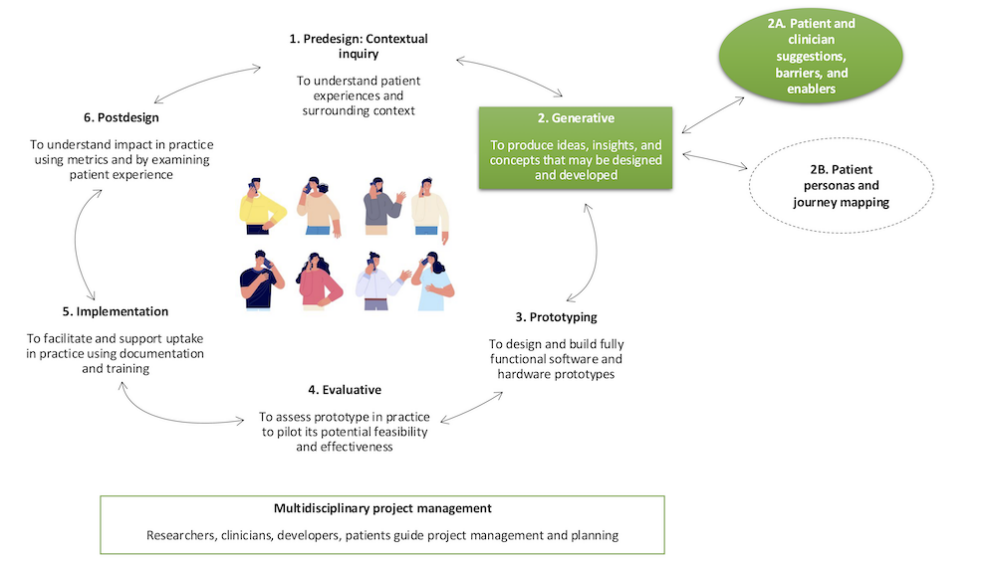
Project co-design stages. The generative phase (phase 2) reported in this study is denoted by the items highlighted in green.

A co-design approach [9] was used, applying the framework by Sanders and Stappers [30] and updated by Noorbergen et al [31]. It outlines 6 iterative co-design phases: predesign, generative, prototyping, evaluative, implementation, and postdesign. Figure 1 presents an overview. This study reports on the generative phase of the iterative co-design development process, which includes exploring barriers, enablers, and suggestions for the digital BI from both the patient and health professional perspectives. Patient perspectives were collected from individual interviews (n=18), and 2 focus groups of patients (n=7) invited to attend after the individual interviews; health professional perspectives (n=5) were collected from two focus groups. All individual patient interviews were conducted via telephone by RAE, a clinical psychologist; KB, a psychologist; and SP, all with expertise in chronic pain or qualitative research. Field notes were recorded during the sessions.

The project was led by the study’s chief investigator (RAE), who is a clinical psychologist–researcher. The multidisciplinary project team consisted of health care researchers (ie, psychologists, physiotherapists, pain medicine specialists, and addiction specialists); digital development partners (ie, software developers and designers); and patient partners (ie, individuals with a lived experience of CNCP including those using prescription opioid medications). The team met weekly (sometimes more often) during the development process.

### Measures

#### Web-Based Questionnaires

Patients were asked to provide their demographic details, information about their pain condition, current medications, and history of mental health and substance use disorders. Validated measures included the Brief Pain Inventory [32,33]; Kessler Psychological Distress Scale [28]; 21-item Depression Anxiety Stress Scale (DASS-21) [34]; and Current Opioid Misuse Measure (COMM) [35]. A full description of the measures can be found in the study by Elphinston et al [8].

Health professionals completed a web-based survey containing questions about their demographics, clinical background and experience, workplace setting, training and professional development in pain, use of DHIs, and types of telehealth use in clinical practice.

#### Patient Semistructured Interview and Focus Group Questions

As part of a larger set of interview questions, patients were asked about how they visualize their pain management in the future and their opioid use and how they could assist others in using opioids more safely. Multimedia Appendix 1 provides a summary of the questions. Patients who then expressed further interest in co-designing the BI were invited to participate in the focus groups, which involved discussing their impressions of the digital BI and several activities exploring its components. To set the stage, participants completed an exercise involving sharing their pain story, including the number and type of pain management strategies used. Participants then completed activities such as questionnaire assessment and feedback, introduction to the biopsychosocial model of pain, opioid use education, personality-targeted coping skills training, and motivational interviewing and action planning. Patients also explored the problem through a “peel the onion” activity and proposed solution requirements and were provided an example of a BI for smoking cessation. The aim of these activities was to explore and confirm patient preferences and needs, drawing on the results of the individual interviews to guide discussion. The role of technology in delivering BIs was explored including how to best meet the patient needs of this population and the implementation requirements. Finally, patients completed a value sliders exercise as a group, rating each BI component derived from research on BI treatment models and patient experience, from most important to least important.

#### Health Professional Semistructured Focus Group Questions

Health professionals were first introduced to the background and rationale for the digital BI, the main components, and the themes identified in patient lived experiences reported in a previous study [8]. They were then asked about their impressions of the themes and the digital BI. Multimedia Appendix 1 provides a summary of the questions.

### Procedure

Interviews and focus groups were completed from June to December 2020. Patients completed an initial telephone screening interview to determine their eligibility for individual interviews and health professionals expressing interest via email were invited to 1 of 2 focus groups. Eligible patients and health professionals completed a web-based questionnaire before the interview for descriptive purposes and to provide background to guide the interview. Patient focus groups were then conducted in person on-site at the clinic with one patient participating via Zoom (Zoom Video Communications, Inc). Health professional focus groups were conducted via Zoom. Interviews and focus groups were audio-recorded and professionally transcribed verbatim.

### Data Analysis

An iterative approach to data analysis was used. Transcripts from the patient interviews and focus groups were thematically analyzed separately from health professional focus group data using grounded theory [36]. Two coders (RAE and SP) coded line-by-line and then grouped the codes into subthemes and themes. Theme refinement was facilitated by a third clinician-researcher (KB), who assisted in integrating interviewer field notes to aid interpretation and reduce coder bias. All researchers were asked to review the themes and subthemes. The audio recordings were referred to as needed to examine patient quotes and clarify content.

### Ethical Considerations

Ethics approval was obtained from the Metro South Human Research Ethics Committee (HREC/2020/QMS/60695). All participants provided written informed consent. Study data were deidentified for analysis, and any identifying information was removed before publication including patient numbers to ensure complete anonymity. All participants were offered an Aus $50 (US $33) gift voucher for each session attended to thank them for their time.

## Results

### Characteristics of the Patient and Health Professional Samples

Patient characteristics (n=18; 55% women; mean age 49.5, SD 6.91 y) are presented in a previous study [8]. We provide an overview in Table 1; for a detailed summary of each individual, please refer to a previous report [8]. In the sample of patients interviewed, half (9/18, 50%) met the threshold for current unsafe opioid misuse on a validated psychometric scale (COMM [35]). Most health professionals (n=5; 40% women; mean age 40.2, SD 5.97 y) were psychologists working in both the private and public sectors, with an average of 9.8 (SD 5.97) years of clinical experience (Table 2).

**Table 1:** Patient (n=18) demographics and clinical characteristics.

Characteristics		Values
**Gender, n (%)**		
	Female	10 (55)
	Male	8 (45)
**Ethnicity, n (%)**		
	Australian	14 (77.9)
	British (English, Welsh, or Irish)	2 (11.1)
	New Zealander	1 (5.5)
	Greek	1 (5.5)
**Pain location, n (%)**		
	Head or face	4 (9.5)
	Neck	6 (14.3)
	Shoulder or upper limbs	6 (14.3)
	Back, spine, or sacrum	12 (28.6)
	Lower limbs	4 (9.5)
	Whole body	5 (11.9)
	Abdomen, pelvis, or groin	5 (11.9)
Age (y), mean (SD; minimum-maximum)		49 (11.5; 25-62)
Pain duration (y), mean (SD; minimum-maximum)		11.6 (11.5; 0.5-42)
Pain intensity (past week), mean (SD; minimum-maximum)		5.8 (1.5; 2.3-8.0)
Pain interference (past week), mean (SD; minimum-maximum)		5.8 (1.9; 2.7-9.1)
Kessler Psychological Distress Scale, mean (SD; minimum-maximum)		9.7 (4.8; 4-18)
**Depression, anxiety, and stress^a^, mean (SD; minimum-maximum)**		
	Depression	10.8 (9.1; 0-30)
	Anxiety	9.2 (8.9; 0-34)
	Stress	13.9 (9.1; 0-32)
Opioid use duration (y; n=16^b^), mean (SD; minimum-maximum)		8.0 (8.7; 0.6-26)
OME^c^ (n=13^b,^^d^), mean (SD; minimum-maximum)		114.2 (166.8; 1.2-480)
COMM^e^ (n=16^b,f^), mean (SD; minimum-maximum)		11.7 (7.89; 1-27)

^a^DASS-21: 21-item Depression Anxiety Stress Scales.

^b^2 participants were not currently using opioids.

^c^OME: oral morphine equivalent.

^d^3 participants currently using methadone.

^e^COMM: Current Opioid Misuse Measure.

^f^8 participants scored ≥9 indicating risk of opioid misuse [35].

**Table 2:** Health professional demographic and background information.

	Sex	Age (y)	Profession	Years of practice	Work sector
HP^a^1	Male	44	Psychologist	17	Public and private
HP2	Female	29	Psychologist	3	Public
HP3	Female	39	Psychologist	15	Public
HP4	Male	42	Nurse	8	Public
HP5	Male	47	Psychologist	6	Private

^a^HP: health professional.

### Patient Preferences

Guiding content, principles, and processes related to the proposed digital BI emerged from the individual patient interviews and were further refined in the patient focus groups. There were 5 themes related to the content of the BI (themes 1-5) and 5 related to the process and guiding principles (themes 6-10). Table 3 provides a summary of themes and exemplar quotes. Patient numbers were removed before publication to ensure anonymity (PID_P refers to patients from the pain clinic and PID_A refers to patients from addiction services).

**Table 3:** Themes identified from the individual patient interviews and focus groups

Themes	Brief description	Key examples from patient interviews
**Intervention Content**		
1. The relevance of the biopsychosocial model and need for improved awareness and pain psychology education	The biopsychosocial model provides a relevant framework for understanding pain processes and improved awareness and pain psychology are needed.	“It [biopsychosocial model] does make sense because I never connected it until I actually spoke to you on the phone. Because I had a good think about it and when there were family issues or when we moved house or anything really stressing my back pain would flare up. and it was almost like my pain levels, like my back was dealing with the stress. I had no control over that, I was just feeling what was going on and the pain levels go up... it’s interesting, I didn‘t know that stress impacted and now when I think way back to when my back pain really kicked in it was a really stressing time.” (PID_P). “I can definitely relate to what you were talking about earlier that even when you’ve recovered your body still holds onto the pain because I feel that that’s very much what’s happening to me... long term pain puts stress on your nervous system and you can hold your pain because I’ve noticed lately that ever since I’ve had this when I get shoulder pain or something like that even just muscular pain it stays for a very long time because my body is in this constant state of alertness that it just holds pain. So, if I get a sore neck or something like that instead of it going in two days which it would 5 years ago, it now stays for weeks or even months because of my like nervous system” (PID_P).
2. Nonpharmacological treatments and flexibility when applying coping skills training are needed	Pain management strategies not just strategies to manage opioid use are required and should be flexibly applied.	“I’d rather just not use the opioids at all and get help in other ways like ether it be therapy...I think Drs should be careful in prescribing them...and look at other things they could do for you” (PID_P). “But if you’re suffering obviously mentally, it kind of puts your body through a bit of stress, stressful situation that can cause like tension or psychologically it will inflict pain...So who knows, maybe a psychologist or whatever that was in pain, to help them mentally think about their pain in a different way, that would probably help...(PID_A).Yeah because you like to be told what to do and I like to choose what I do. But that’s different personalities. I’m the boss of me, no body be telling me what to do, you know” (PID_P).
3. Opioid use reflection and education, with personalized medication and tapering plans	Opportunities to reflect on if opioids are working for them and providing the necessary education as well as medication plans that are individualized to the person’s circumstances.	“Explore potential problem in a non-judgemental way... I think finding out what the reason is and why it is important... But I think it’s very important for the doctors, as the ones prescribing the medication to actually sit there and go, all right, here’s the side effects” (PID_A). “Refer to someone who can help taper in a personalized way... clear taper program with options to increase dose again if needed ... More regular monitoring by GP e.g. every month instead of 6 months” (PID_P). “They want to come off them, if they think that they no longer have a physical use for them and might only be psychological they would need something to help them get through withdrawal symptoms” (PID_A). “I don’t feel the GP’s support is enough...GP never asks me to keep a diary...they are not actually checking on if you have taking too many” (PID_P).
4. Holistic and patient-inclusive assessment measures and feedback	Assessment measures & feedback are holistic and patient inclusive.	“I like how it [COMM] asks a lot of questions about how you feel and relationships like arguments... because I think pain effects so many things. It effects your relationships. it affects how you communicate with people. If you’re in a lot of pain you can’t think clearly. You are and you get angry at anything you get ticked off or annoyed or frustrated. So I think a lot of these comments if you know people are in pain they could relate to this very [sic] a lot of these questions...very relevant.” (PID_P).
5. Inclusion of holistic goals targeting comfort and happiness	Broader, personal goals that target the whole person should be included, with a specific goal to increase happiness and level of comfort rather than a focus on being pain-free.	“[Success would look like] A happy person... a well-adjusted person. A person who’s not in constant pain. The list goes on... I would be happy with it making me comfortable” (PID_P). “[Facilitator: not just medication focused goals?] Because I think it all comes to, its holistic, it’s all encompassing, every part of your life needs to be looked at [– group agreed]” (PID_P).
**Intervention Process and Guiding Principles**		
6. Therapist-guided when needed and accountable	Guidance from health providers preferred rather than self-management approach to facilitate patient motivation and ensure accountability.	“Could lose motivation very quickly if doing it yourself” (PID_P). “Maybe having a drug counsellor or someone they can speak to on phone or online when they feel they are struggling with their medication” (PID_P). “Psychologist to your house and the house visits for people that can’t get out. That would be ideal, you know... Video for a psychologist would be good... I just get all tense to probably just when I’m in front of someone talking to, but that’s just me” (PID_A).
7. Engaging easy to use features including peer support	A combination of engaging features (visual, verbal) including peer support to share similar experiences and ideas with and provide moral support when needed.	“Even just hearing everyone’s story, just feel not as alone” (PID_P). “Sponsor like AA where if they want to take an extra tablet, they talk you out of it” (PID_P). “Someone I could talk to, relate to, be there, just for the psychological side of it. People to talk to... Moral support was a big part of it. I reckon if I had someone to talk to a lot, when I was going through it wouldn’t have been so bad. Just having friends that are going through the same thing” (PID_A).
8. Compassionate, responsive and person-centered care	Treat the person not the number, providing nonjudgmental and care personalized and responsive to the patient’s needs.	“I would have appreciated at the start not being told it was all in head and having just opioids thrown at me” (PID_P). “Support, having someone see them as a person and not see them as a number” (PID_A). “It’s not a one size shoe fit all [approach to pain management]” (PID_A). “With our other doctor, at least we could drop in and say, oh, my God, it’s freaking hurting today” (PID_P).
9. Digital solution was new and exciting, maximizing reach	The new and exciting option of digital brief interventions maximizes reach in a variety of settings (eg, primary care; specialist wait lists).	“If there was sort of like a program available and then that was part of it, so if I ever went to the doctor and said I want to reduce my tramadol slowly over time he could say “well, here’s the website” and he’ll register me for it and sort of he communicates with that website as well so when I go back to him he can see that I’ve done all the criteria and then he can reduce my medication” (PID_P). “It would be really really good to be able to have something where you could be like, what can I do now? That would be really good because then you feel someone is on your side, someone is trying to help you plan. You’re not just being left” (PID_A).
10. Educate and normalize challenges navigating the health system and policy	Education about policy changes and normalizing the challenges of navigating the health care system.	“The GP put me on tramadol in 2007 and when the government brought that thing on the first of June, I had a new doctor and all of a sudden it’s like oh no you can’t be on this, you’ve got to be off, and I’d actually just switched to a new GP and he thought I was doctor shopping. He was quite judgmental” (PID_P).

### Intervention Content

#### Theme 1: The Relevance of the Biopsychosocial Model and Need for Improved Awareness and Pain Psychology Education

Participants agreed that the biopsychosocial model of pain provides a relevant framework for explaining the unique individual experience of pain and how it can change over time. The patient experience interview was the first time when 3 of the 4 focus group participants were introduced to this model [8].

Most participants reported a number (>10 types) of varied biological and psychological influences of pain. It was important for participants that the origins of pain were explored (eg, surgery, injury, gradual or unknown, and stress). For another participant (PID_P), the continuation of pain despite recovery from physical injury resonated. One participant (PID_P) reported that they had moved toward acceptance that pain was mostly perpetuated by psychological factors including stress and tension as they had eliminated all other reasons for their pain; they were now using mindfulness, breathing, and monitoring their own posture to relax and manage pain after having ceased the use of opioids in the past 8 weeks (a significant change from the individual interview [8]).

Participants also reported that the concept of a person’s personality characteristics contributing to their pain and opioid use was considered important (eg, “Are you a 100-mile person or are you slow and steady, you know, it misses that completely” [PID_P]). Most participants reported that they identified with both depressive- and anxiety-prone personality characteristics as contributing to their pain and opioid medication use.

Social factors were considered less influential. One participant described how the COVID-19 pandemic had impacted their ability to go to church and that church often resulted in feeling better in terms of her overall well-being and pain—“I feel better when I come out of church then I did when I go in, and you know pain wise and all over wise” [PID_P]. Another discussed the role of caregiving responsibilities on her pain management experience—“[social factor] well I can’t try medical marijuana even though a doctor offered it to me because I said to him I have to roll out of bed at 2 in the morning if her [elder mother who they care for] alarm goes off so I can’t be taking that” [PID_P]. This facilitated patient understanding of how individualized social factors can influence pain experience.

Participants reported that greater awareness and educating patients about the biopsychosocial model of pain is needed. One participant (PID_P) described how it was helpful to understand how pain impacts the nervous system over time and how the body “holds” pain, especially because their injury had healed, and they now wanted to understand more about the influence of mental health. Another participant was open to discussing the role of mental health in pain with psychologists or psychiatrists because their injury had gone, and pain was persisting (PID_P).

#### Theme 2: Nonpharmacological Strategies and Flexibility When Applying Coping Skills Training

Participants confirmed a need for nonpharmacological strategies other than the use of opioids such as relaxation, exercise, engaging in pleasant activities, and pacing. PID_P said, “there’s got to be something out there that covers everything but doesn’t actually interfere with life itself, because you know the opioids make you stupid.” This was reported by some participants as important to “take back control” (PID_P) from doctors who “should stop giving out opioids and recommend other non-pharmacological alternatives” (PID_P). One participant (PID_P) wished not to be opioid dependent, as they feel reliant on pain medication:

If [the BPI could] teach you not to be drug dependent. Because that’s pretty much what I rely on. If that just stopped, I don’t know what would happen.

There were a range of coping strategies that participants identified could target depressive-proneness and pain (eg, listening to music, deep breathing, and doing enjoyable things). Coping strategies targeting anxiety-proneness included relaxation and releasing tension in the body (eg, gentle stretching or yoga) and mindfulness and meditation, while additional strategies that could target both personality styles were discussed (eg, going for a walk or talking to someone about how you feel). Multimedia Appendix 2 provides a snapshot of this exercise.

It was important that there was flexibility when applying a personality-targeted approach to coping skills training; some participants liked the targeted approach and wanted to be directed to the strategies corresponding to their personality style. Others preferred to be offered a full range of options and an opportunity to select the strategies themselves appealed to them:

Yeah because you like to be told what to do and I like to choose what I do. But that’s different personalities. I’m the boss of me, no body be telling me what to do, you know.

PID_P

I don’t know if the selection will work because if you’re feeling anxious and stressed, being told to pick one of them is going to be overwhelming itself. You know if you’re lonely and depressed, you might not necessarily want to choose you might just want someone to say right you’re doing this. Like a decision might actually contribute to this. [all agreed with this]

PID_P

#### Theme 3: Opioid Use Reflection and Education, With Personalized Medication and Tapering Plans

Some participants reported that opportunities to reflect on their response to opioid therapy (benefits vs harms) would be important and could offer an avenue to provide opioid use education. Patients indicated that more knowledge of the potential harms of opioids was needed. One participant (PID_P) described how education that opioids can increase pain sensitivity and that tapering opioids do not necessarily result in increased pain was helpful when reducing opioid use:

I never attempted to come off it because I just thought what am I going to do with my pain, it’s just going to get worse and he said no, as you reduce your pain sensitivity will reduce and he was right...[knowing that] it took the fear away from tapering off.

Related to theme 7, the use of trivia and quizzes as a regularly updated semicompetitive activity was suggested to engage patients in education and motivation to continue with the digital BI.

Some participants suggested that education about opioid addiction and dependency would be important as well as taking into consideration people with pain who may have had a history of substance use problems. One participant said, “I’m clean. and even when I broke my shoulder and I had to take fentanyl in the hospital I phoned my sponsor first to make sure that it was okay for me to have it” [PID_P].

There was a specific need for opioid medication management plans with a focus on medication-related goals that are achievable:

Reduce your dose by I don’t know 2mg, that’s achievable... just little things you know make sure you go for a 10min walk once a week...10mins once a week should be achievable and not overdo it. You know just little things, it’s like the compound effect book... you make such a small change that you don’t really notice it but its compounded over 12 months... small achievable goals that compound.

PID_P

For some people, goals were related to a reduction in medication dose, while for others, using medication more safely. Opportunities to revise goals over time (eg, weekly) was identified as essential. Participants discussed a tailored, stepped, and “SMART” (Specific, Measurable, Achievable, Relevant, and Time-Bound) approach to goal setting. Monitoring of personalized medication plans was reported by some participants as necessary to checking patient response to treatment and provide an opportunity to identify any concerns (eg, withdrawal). One participant (PID_P) suggested a patient medication diary could assist in the monitoring process. Collaboration among health professionals and with patients was highlighted to ensure that everyone is aware of the patient’s individual medication plan and is working together to support the patient’ goals.

#### Theme 4: Holistic and Patient-Inclusive Assessment Measures and Feedback

Participants reported that assessment measures could focus on pain, its impact, medication use, and personality styles. It was important to all that measures are inclusive in terms of language and are nonjudgmental. In presenting the assessment measures to participants that they completed before the study, participants reported that they liked how items on some measures asked about how you feel (eg, capturing feelings of anger and frustration) and the impact of pain on relationships (eg, arguments assessed by the COMM).

Participants reported that they did not think the measures presented captured the pain journey (in terms of historical factors) as well as personality factors, although the research team communicated that this was probably a function of only providing participants with opioid-related measures. Focusing on opioid use in the past 30 days (as noted in the COMM) was viewed as restrictive:

This is all in the past 30 days, yet it says holistic there [on whiteboard], peoples pain experience, you know a holistic pain experience isn’t just in the last 30 days it’s in the last years...people have ups and downs, you might have had a really great 30 days. It doesn’t really represent somebody’s holistic pain experience.

PID_P

The items were reported to result in some participants feeing judged (eg, taking opioids illegally or outside of their prescription and counting pills) and that they were a “drug addict” [PID_P]. Participants discussed a sense of relating the pill counting questions more to anxiety than misuse:

[SOAPP-R] counting pills, only reason I would count them is because I’m anxious about not having enough, not because...its anxiety related not accumulative.

PID_P

It was suggested that the wording of some of the items could be modified to be less stigmatizing or measures could be introduced to participants and sensitivities flagged. Asking participants how they felt about questions about opioid use and giving them a free text box response option could also be beneficial (PID_P).

#### Theme 5: Inclusion of Holistic Goals Targeting Comfort and Happiness

Participants suggested that goals should target the whole person in addition to focusing on medication action plans. One participant (PID_P) discussed that they felt able to focus on improving physical fitness in the context of having an achievable plan already in place for reducing her tramadol use and managing withdrawal symptoms. It was also discussed that goals should empower patients, facilitating a sense of control and autonomy, consistent with a biopsychosocial mindset (theme 4; eg, success=confidence+mood boost=reduced pain):

Once you achieve something and you’ve got that little bit of success, that endorphin release also helps with your pain. [PID_P said “yeah yeah”] So, you’ve achieved something and that feel good feeling is yeah.

PID_P

Specifically, the goal to increase happiness and level of comfort rather than a focus on being pain-free was proposed as a possible goal of the intervention identified during the “peeling the onion” exercise (to gradually work toward deeper understanding of the problem to be solved). Participants suggested that focusing on making your life better and to increase happiness may assist in helping people to lead a “normal” lifestyle. Interestingly, participants did not focus on the goal of being pain-free but rather on being “comfortable.” Most had moved toward acceptance of pain and felt that they may never be pain-free. Multimedia Appendix 3 provides a summary of the exercise.

### Intervention Process and Guiding Principles

#### Theme 6: Therapist Guided When Needed and Accountable

Participants highlighted opportunities for connection and support from health professionals and peers were important. There was a need for “good advice” when called on and someone to talk to on bad pain days. Resources (eg, website links) on topics of interest were also viewed as helpful. A chat box feature was suggested as one way to connect with health professionals. Immediate responses from health professionals and options from where to seek further help were also seen as important (eg, drug and alcohol phone service). Participants agreed that the BI needs to be therapist guided rather than a self-management approach. It was suggested that a therapist-guided approach would provide both extra support and also accountability: “You’d want it to feel like there is that actual support” (PID_P); “I guess if it’s not being monitored then you think what’s the point?” (PID_A). Participants discussed value in health professional contact at the initial onset of the BI, in completing assessments and in the pain journey discussion.

#### Theme 7: Engaging, Easy-to-Use Features Including Peer Support

Discussions on how to best engage patients in the digital platform included the consideration of color schemes, the use of games and competitions like “scoreboards,” and the “steps challenges,” which were viewed as motivating. Reading and sharing of patient stories would help normalize the patient’s experience and provide ideas for what has worked for others. PID_P described the value of Facebook support groups. Hearing others’ stories were highlighted as important: “Even just hearing everyone’s story, just feel not as alone” [PID_P]. There were examples of chat boxes, website links, chat rooms, and forums to facilitate this, and it was suggested that this support may be very helpful, particularly on bad pain days. One participant suggested that access to journal articles that were translated into simple summaries for patients or videos of user-friendly, up-to-date evidence-based research summaries would be helpful. It was important that the BI is simple and easy to use.

#### Theme 8: Compassionate, Responsive, and Person-Centered Care

Participants reported a need for compassionate and personalized care from all treating health professionals. It was important that they were seen as a person and not as a number (PID_P) and that care is tailored to each unique individual with pain. Comments about “being told pain was all in my head” [PID_P] were viewed by participants as invalidating and unhelpful to treatment discussions. Empathy was agreed as a necessary ingredient to care, as was the responsivity of health professionals. Some participants suggested that greater access to health professionals, particularly on bad pain days, via digital means could better meet their support needs (eg, “GP contactable on Facetime, he does tele[health], emails” [PID_A]).

#### Theme 9: The Option of Digital BIs Was New and Exciting, Maximizing Reach in a Variety of Settings

None of the participants had come across a digital BI for CNCP in their search for effective treatment solutions. They said they were excited for a new option that could potentially help themselves and other patients. Participants discussed hope that their experiences would inform the design and development of the BI that could offer more support and earlier intervention to patients. A digital platform was seen as an ideal way to deliver the intervention for maximum reach and access in a way tailored to the patient’s needs. One patient had experience using apps (eg, Smiling Mind), which was reported to give them more confidence in the potential use and acceptability of a digital BI.

Participants suggested that disseminating or promoting the BI would be suited to a primary care setting, with the use of flyers in the waiting room or to those on specialist waitlists. Engaging potential participants in the intervention could also include the use of social media, Google advertising, and BI advocates and word of mouth. Since it was discussed that everyone is generally using their phone on a daily basis, there was agreement that web-based promotion may be more effective than hardcopy leaflets. Health professional and particularly general practitioner education and awareness of the BI program was seen as key.

#### Theme 10: Educate and Normalize Challenges Navigating the Health System and Policy

Participants discussed difficulties navigating the health system and indicated value in providing education on the rationale for policies and legal restrictions that may impact their opioid use and pain management. It was suggested that greater consideration of the social environment and medicolegal background may serve the function of normalizing challenging experiences (eg, changes in opioid use), acknowledging differences in the treatment they received in the past (20 years ago) compared to current best practices, and reducing perceived judgment from the care team (eg, methadone associated with feeling judged as “a drug addict”).

### Patient Ranking of Importance of Intervention Components

Patients ranked the importance of 7 digital BI components from most important (rating=1) to least important (rating=7):

1. Support
2. Coping strategies
3. Education
4. Assessment
5. Feedback and monitoring
6. Goals and action plans
7. Fun

### Health Professional Perspectives

Five themes were identified from the data as important considerations by health professionals to the development of the digital BI and its implementation in clinical practice. Barriers and enablers were also identified.

#### Theme 1: Use of DHIs and Resources Within Clinical Practice Was Rare But Desired

Health professionals described that they generally do not tend to integrate apps and digital interventions into their clinical practice. One health professional said, “it would be rare that I’d use a program in the clinic with someone I’m actually treating.” Instead, they were generally viewed as an adjunct to treatment. For example, health professionals would recommend web-based programs for pain or more general mental health apps. Health professionals also recommended patients’ access TED Talks, podcasts, and educational videos, as well as resources they had developed themselves (eg, mindfulness audio files and sleep hygiene information). Most only recommended resources that they have evaluated themselves. Some health professionals highlighted that integration of digital health tools into their practice was practically challenging: “I’ve never really tried to integrate an app into treatment because it’s always been a bit too challenging in terms of working out how to get the feedback and what to do with it then.”

There was recognition among health professionals that it is important to point patients to digital resources that are free and easily accessible: “They are not all free, or only free for a while and then a subscription is required.” It was also acknowledged that some patients may not have access to the internet and that digital resources were generally not inclusive of culturally and linguistically diverse patient populations. Some health professionals reported that they would like to use digital health resources more in their clinical practice: “Depending on the demographic as in CALD [culturally and linguistical diversity], not really many resources I can use but I don’t use it as much as I would like to.”

#### Theme 2: Digital Health Useful for Patient Monitoring of Symptoms and Access to Support on the Run

Health professionals reported that apps can be useful for patients to monitor their symptoms over time as well as track their use of other treatments (eg, cannabis). There were reports that digital health tools can increase access to support such as coping strategies and resources. One health professional said, “Can be useful to access it remotely such as if they are in a shopping center and feel anxious they can sit down and do some calming exercises.” While benefits were mentioned, some health professionals reported that their clients preferred to minimize the use of digital health: “Many of my clients just prefer to keep it simple, won’t even use video [during telehealth], just want a phone call consultation.”

#### Theme 3: Patient Motivation to Use Digital Health Resources Is Important

Patient motivation to use digital health was considered important in whether these were successful in clinical practice. One health professional said, “I find that that apps are only as good as the client’s motivation to use them,” and another said, “Sometimes we recommend things and they don’t get acted on...take a lot of prompting to use themselves.” There was acknowledgment that patient motivation can be influenced by several factors (eg, how easy it is to download an app) and that some patients may not be motivated to change behavior, try something new, or use digital health resources.

#### Theme 4: A Digital BI App for This Complex Problem May Be Beneficial at Multiple Points in the Health Care Journey

Overall, health professionals agreed that a digital BI for the complex problems of CNCP and risk of opioid-related harm may be beneficial for patients. Health professionals reported that the digital BI could be helpful for patients to complete before referral to pain specialist services:

I see it as helpful before come to me [specialist pain clinic] because it will shift their readiness for change and make more open to when they come to me. Would use this as a pre-intervention.

There were also reports that the digital BI may be “Useful to bridge gap between primary care and pain services, so not waiting for so long.” One health professional said the following:

Good for those on the waiting list for specialist services... it might mean that by the time they get to that service they might be more ready, knowledgeable, more open to alternatives... help to set up expectations of treatment approaches.

Others reported that it could fit within a primary care setting or even be used for the prevention of opioid use problems:

I think it could be useful across the board. In my clinic though, patient selection is important if you want to get best results. Could help to move people who are at the pre-contemplation stage.

The patient’s stage of change or motivation was reported as important in considering the best intervention point (linked to theme 3). There was also suggestion that the digital intervention could be delivered by psychologists working in the private sector; mental health professionals; multidisciplinary pain clinics; or, in the primary care setting, by nurses or physicians: “the GP could refer patients to or deliver the app before they prescribe.”

Health professionals reported that patients would prefer to access the intervention via a mobile app or device rather than a desktop website due to convenience and portability. There was also recognition that a digital intervention would have challenges for some patients, and there was a need for therapist-guiding:

Both (app and website) have access issues. Would be good to have an option to do the program somewhere; in the GP office, at the MDT [multidisciplinary team] clinic while they’re waiting on the wait list etc. Either way patients would need health professional support for access and troubleshooting as well as for the monitoring and goal setting.

#### Theme 5: Realistic Patient Goals Focused on the Safe Use of Medications and Managing Pain

Patients’ goals that are focused on safe use of opioid medications including increased willingness or openness to change unsafe use were supported. Health professionals also reported that goals related to use of alternative pain management strategies and pain acceptance may also be achievable: “Can we get to them to move a little bit on changing behavior - willingness to change opioid use and use alternative strategies.” Setting realistic goals were important:

I’d work on positive wins towards the overall goal. So much going on for patients, other things to manage.

### Barriers to Implementation

Some health professionals reported that more than 4 hours of intervention may be required to achieve the patient’s goals. Costs related to who would deliver the intervention were identified as a barrier as well as clinicians setting aside time for support (eg, viewing patient monitoring). There was also concern that some patients may be defensive about making changes to their opioid use and that some patients do not trust health professionals to talk about their opioid use.

### Intervention Enablers

Health professionals viewed that the underlying philosophy of the digital BI (motivational interviewing and enhancing patient self-efficacy) was consistent with how they approach their clinical work. There was recognition that implementation of the digital BI would be most effective if considered within a stepped-care model (ie, primary care > digital BI > specialist care; related to theme 4). There were also reports that feedback to patients (about their opioid use, pain, and mood) is “always helpful,” as is patients’ response to this information. This information can then be used to troubleshoot or problem solve.

## Discussion

### Main Findings

**Figure 2:**
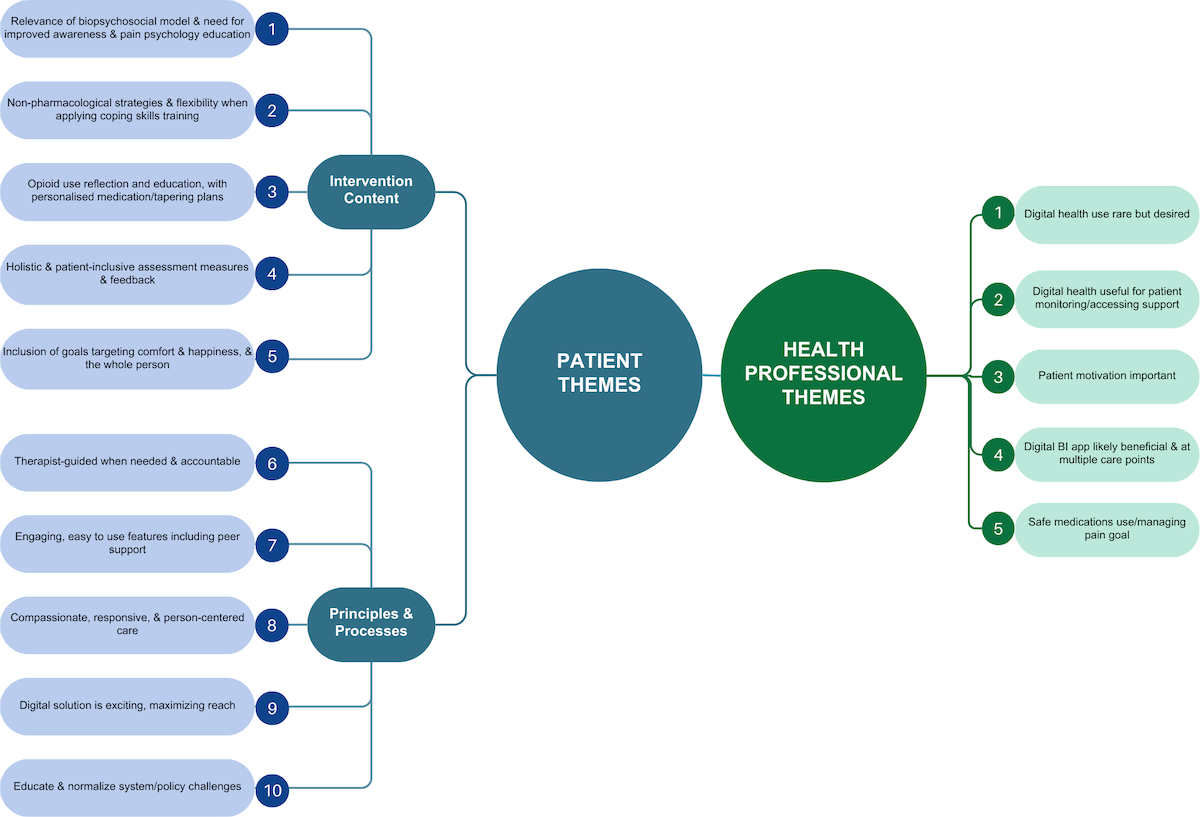
Snapshot overview of themes.

This study aimed to understand patient and health professional preferences, including implementation considerations, for a new digital BI to reduce prescription opioid–related harm among patients with CNCP. In total, 10 themes, 5 related to intervention content and 5 related to intervention processes emerged from patient reports as important in the future digital BI co-design and co-development. From reports of experienced health professionals, we identified 5 themes, as well as several barriers (including intervention intensity, potential costs, and patient responsiveness) and implementation enablers (including consistency of theoretical underpinnings with current practice, patient feedback, and the digital BI as part of a stepped-care model). Figure 2 provides a snapshot of the themes identified from both patients and health professionals.

Overall, both patients living with CNCP and health professionals were interested in the proposed digital BI to reduce opioid-related harm. Health professionals highlighted that digital health tools may be particularly useful for patient symptom monitoring and access to coping skills support (eg, deep breathing exercise) when on the move. Patients agreed that a digital solution could expand the reach of BIs to people who need it. Certainly, there is growing interest in and evidence for the role of digital BIs in treating pain [13,37-39].

In line with clinical guidelines that recommend multidisciplinary and behavioral treatment approaches as first line (eg, Dowell et al [40] and Busse et al [1]), patients reported that the inclusion of nonpharmacological pain management strategies (eg, relaxation, exercise, pleasant and valued activities, pacing, access to psychology, and multidisciplinary team approaches) would be welcomed, rating this as one of the most important features of the digital BI. Patients also indicated that personalizing coping skills training to patient personality characteristics (eg, impulsivity traits) and pain-related factors (eg, depression) could benefit some patients, while others may prefer to direct themselves to coping skills that resonate with them. Individual choice could be built into the digital BI to recognize patient differences in autonomy and self-efficacy. Notably, nonpharmacological approaches can achieve similar or even greater improvements in pain and functioning without the potential harms of dependence, adverse side effects, and overdose. This is especially important as patients generally report that opioid therapy has modest effects on levels of pain and functioning [41-43].

There were educational needs identified by patients in the areas of pain psychology, the biopsychosocial model of pain, and opioid therapy. These findings are in line with the high levels of interest and increased need for patient education about the role of psychology in the management of pain [4,44]. Educating patients about the biopsychosocial model of pain forms a part of multidisciplinary rehabilitation and discipline-specific treatments that can be effective in reducing pain and disability in the short to medium term (eg, Siddall et al [45] and Wood and Hendrick [46]). Helping patients to reconceptualize pain can also enhance their ability to cope with their condition [47]. The importance of safe opioid use education is highlighted in clinical guidelines [1,48]. Few studies have examined the effectiveness of opioid use education for patients with chronic pain. On the basis of the evidence available, opioid-related education can lead to safer behaviors (eg, less stockpiling [49]) and reduced opioid use following surgery [50]. Gaps in educating patients in pain management, influential biopsychosocial factors, and safe use of opioid medications continues to put patients at risk of poor recovery. Ongoing weighing up of the benefits of opioid therapy in the treatment of CNCP versus risks could be facilitated by a health professional–delivered digital BI. This could be embedded within the review of the patients’ personalized medication plan and be supported by regular monitoring and assessment of medication use as well as related symptoms that consider the *whole person* (eg, pain, mood, and functioning). Health professionals considered digital health as an optimal platform to facilitate patient monitoring of symptoms. Patients also reported that one of the top 3 activities that they want to do on their mobile device is manage their medication and track their health [51]. Digital BIs that aim to reduce risk of opioid-related harm have the potential to meet this need.

Patients tended to focus on the potential of the digital BI to meet general well-being goals and reduce pain discomfort to facilitate safer use of opioid medications. Health professionals indicated that patients would be likely interested in pain reduction goals but suggested that goals related to the safe use of opioid medications could also be important. If goals are to encompass the needs of the whole person, then it will be important to explore and balance patients’ pain-related goals and opioid medication harm reduction goals as part of holistic goals related to relationships, work, and lifestyle. To facilitate behavioral change, different innovative approaches (eg, gamification and serious games) could be explored in future studies.

Health professionals reported a desire to integrate DHIs into their clinical practice in a more conscious way. Real-world implementation of DHIs have often failed, mostly because they are not used by patients or health professionals [52]. Engaging health professionals in the co-design process has potential to enhance digital BI feasibility and acceptability in practice, as the success of such measures in health care settings relies heavily on the engagement of key stakeholders [22]. Our results could inform the development of a broader digital BI implementation strategy that considers barriers identified by health professionals (eg, costs, time, and compatibility between the digital BI and their current workflow) and enablers (eg, perceived fit of the digital BI approach to current practice), which are common across DHIs and health care settings [53]. Further consideration of the systems used (eg, mobile app vs website and health professional assistance) and adaptability, policy and guideline support, resourcing, health professional knowledge and beliefs, and involvement of digital BI *champions* to support patient motivation [53] may inform effective digital BI implementation. Most psychologists and mental health professionals (72%) have little or no formal training in pain management and more than 50% report feeling a lack of confidence in treating pain [4]. Training and education for health professionals in delivering digital BIs could fill this gap.

Similar to other digital BI studies [54], a therapist-guided digital BI was preferred among patients and health professionals. According to patients, therapist guidance would enhance patient motivation and engagement and provide more accountability than self-guided digital BIs. Evidence indicates that guided digital cognitive behavioral therapy and face-to-face therapy for psychiatric and somatic conditions produce equivalent overall effects [55], and when therapist support is included, this has a positive impact on engagement and treatment effectiveness [56]. Therapist-guided digital BIs provide opportunities for establishing mutual trust, which can be a barrier to intervention success [57]. This may be critical as health professionals report that discussions about chronic pain management, including medication use, can be challenging, with patients reporting that the context of opioid policy changes can further fuel mistrust. Guided digital BIs allow for therapists to foster a strong therapeutic alliance with empathy and compassion, treating patients as partners in their pain care - providing the foundation that is necessary for treatment success. Further consideration of communication methods between patients, their treating clinicians, and the broader team within the digital environment may further personalize pain services and facilitate multidisciplinary collaboration. When asked to rank the importance of the BI features, patients reported *support* was the most important feature, while *fun* was the least. Future co-design opportunities could explore meaningful ways to integrate gamification and digital peer support to facilitate patient engagement.

Both patients and health professionals reported that digital BIs could be best placed and adopted in primary care, multidisciplinary pain centers while patients wait for treatment, and addiction services where it could be an adjunct to in-person treatment; it was also suggested that it could be tailored to prevent risk of opioid-related harm in people with acute pain. There was also a suggestion that digital BIs could form part of a stepped-care model. Further research is needed to determine whether digital BIs for treatment of risk of opioid-related harm are effective and for whom and in what setting.

### Strengths and Limitations

Most previous studies have not included the views of key stakeholders in the design of DHIs [17,25]. We advanced the co-design of a digital BI to reduce risk of opioid-related harm among people with CNCP by examining both patient and health professional perspectives on their intervention needs and preferences. Our study adds to the growing literature on co-design in DHIs [58] and extends research into the CNCP field.

We included data from 18 individual patient interviews and from 7 patients and 5 health professionals as part of focus group workshops. While there are no guidelines on the minimum number of key stakeholders or frequency and intensity of engagement that is needed in co-design, it is possible that our results may not be generalizable to the broader population of people with CNCP who are taking prescription opioids, or beyond the Australian context where patients have access to a relatively high standard of public health care and access to heavily subsidized medications. Continual engagement of various patients and health professional perspectives in the subsequent steps of co-design and co-development of digital BIs could increase customizability of the solution to fit a wider range of users. In future design and development work, consideration of how to design BIs for better inclusion of patient cultural and linguistic diversity, spiritual needs, and socioeconomic factors is needed, as well as investigations in countries with different health and insurance systems. It will be important that the digital divide does not exacerbate existing health inequities of those most at risk in the CNCP population. Efforts to measure digital inclusion could increase translational confidence in digital BIs.

In addition, there remains a power differential between patients, health professionals, and researchers, and it is unknown how this may have influenced our findings. Future work should explore jointly developed guiding ethical principles to address the relationship power imbalances in co-design. There are also potential tensions between these stakeholders in their digital BI needs and preferences which could be explored in future joint focus groups. Finally, this study was conducted before the widespread introduction of generative AI technologies (eg, ChatGPT, Gemini, and Co-Pilot) to the public. Future studies could explore the integration of generative AI into DHIs, such as those that aim to reduce risk of opioid-related harm and improve pain care.

### Conclusions

Partnering with patients who have CNCP and health professionals to identify their intervention needs and preferences is critical to the co-design of new, innovative, and high-value digital treatments [59]. Patients and health professionals were interested in the proposed digital BI, identifying many content areas, principles, enablers, and barriers to guide the development of such programs. This co-design approach has the potential to enhance the translation of digital BIs into practice and improve clinical effectiveness. The next phases in co-designing personalized digital BIs involve the generation of later design concepts (eg, journey mapping and persona development) and prototyping, while considering the development of an implementation strategy. Continual, genuine bringing together of scientific, patient, clinical, and technical expertise to solution design could address the urgent need for better patient-centered CNCP care while minimizing the risks of opioid medication–related harms.

## Appendix

Multimedia Appendix 1 Patient and health professional semistructured interview and focus group questions.

Multimedia Appendix 2 Personality-driven coping strategies.

Multimedia Appendix 3 Goals identified during the "peeling the onion" exercise.

## Abbreviations

BI: brief intervention
CNCP: chronic noncancer pain
COMM: Current Opioid Misuse Measure
DHI: digital health intervention
SMART: Specific, Measurable, Achievable, Relevant, and Time-Bound

## Data Availability

The datasets generated during and analyzed during this study are not publicly available due to limitations of ethics approval but are available from the corresponding author on reasonable request.
