# Supplementary material for: Applying Patient and Health Professional Preferences in Co-Designing a Digital Brief Intervention to Reduce the Risk of Prescription Opioid–Related Harm Among Patients With Chronic Noncancer Pain: Qualitative Analysis
Source: JMIR Form Res. 2025 Apr 25;9:e57212. doi: 10.2196/57212 (PMC12064972; doi:10.2196/57212)
Supplement: Multimedia Appendix 1 [file formative_v9i1e57212_app1.docx]

**Supplementary Materials**

Table S1. Patient and health professional semi-structured interview/focus group questions.

| Semi-structured interview/focus group questions |
| --- |
| **Patients** |
|  |
| What would you like your pain management journey to look like in the future? |
| What would you change about your pain journey? |
| How would you like your opioid use to be different in the future? |
| What would be the benefits/downsides to making changes to your opioid use? |
| What suggestions do you have for increasing people’s motivation to make a positive change to their opioid use? |
| How do you suggest we help patients to use opioids more safely including programs and interventions that are available? |
| What are the strategies that might change people’s beliefs about pain medication or change their expectations that they will need opioid medications in the future? |
| How would health professionals, services might help people to better manage their opioid therapy? |
| How would we reach those that might be using opioids unsafely? |
| What are your preferred modes for accessing support? |
| **Health Professionals** |
|  |
| What are your initial impressions of the digital brief intervention? |
| What digital resources and programs do you use in your clinical practice? |
| How easy it is to access digital resources? |
| Which patients would best respond to digital brief intervention? |
| When, where and who should deliver it? |
| How would you integrate the digital BI into your practice? |
| What are the barriers/enablers to implementation in practice? |
| What recommendations/other considerations do you have to support the success of the intervention? |
