# Supplementary figures and images for: Applying Patient and Health Professional Preferences in Co-Designing a Digital Brief Intervention to Reduce the Risk of Prescription Opioid–Related Harm Among Patients With Chronic Noncancer Pain: Qualitative Analysis
Source: JMIR Form Res. 2025 Apr 25;9:e57212. doi: 10.2196/57212 (PMC12064972; doi:10.2196/57212)

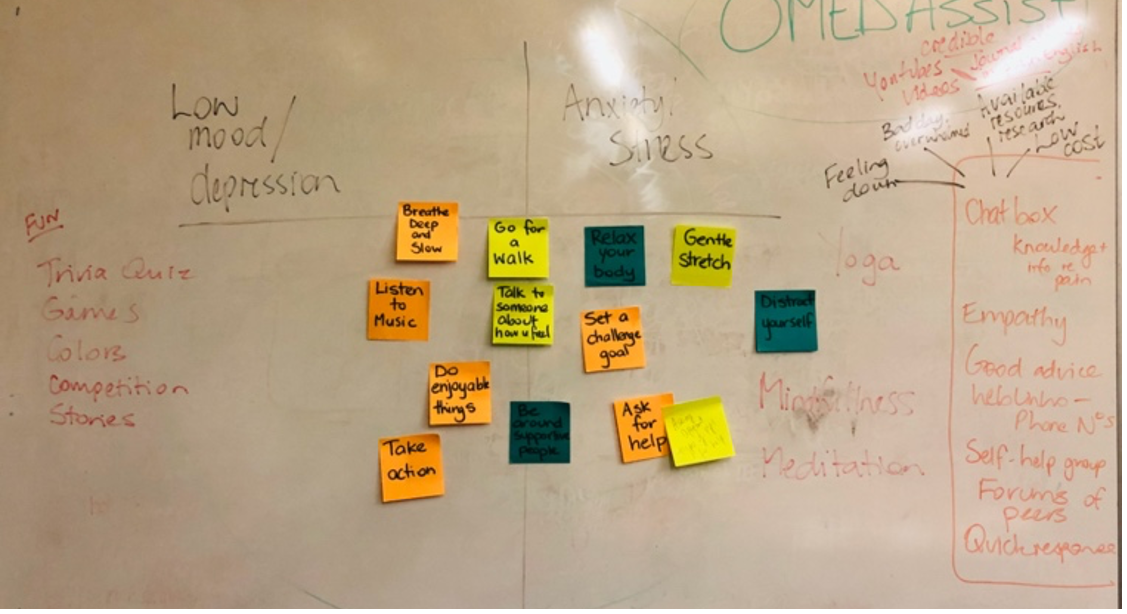

Supplement: Multimedia Appendix 2 [file formative_v9i1e57212_app2.png]

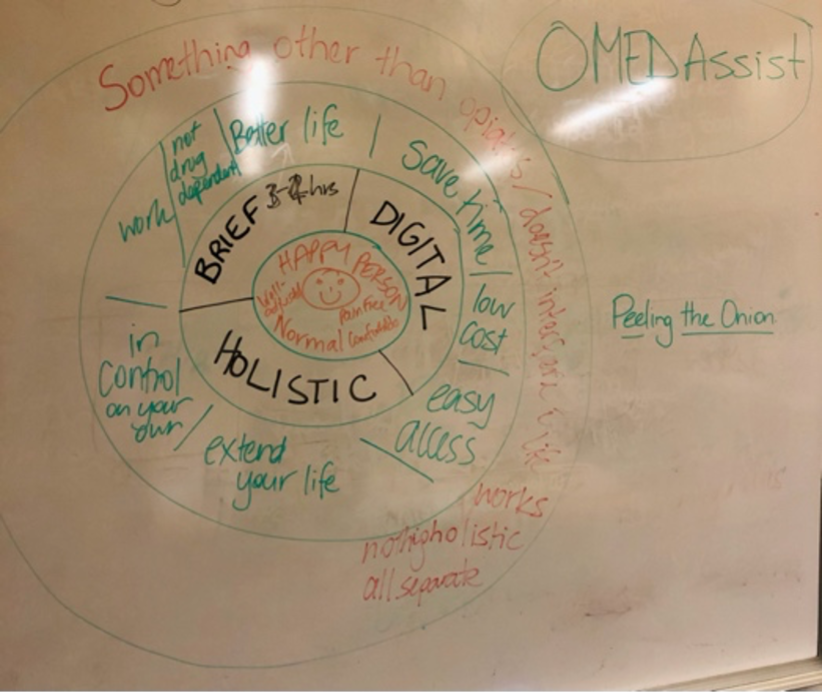

Supplement: Multimedia Appendix 3 [file formative_v9i1e57212_app3.png]
